# A Case of Ataxia in a Child Aged Three and a Half Years

**Published:** 1911-06

**Authors:** Edward Cecil Williams

**Affiliations:** Physician to the Royal Hospital for Sick Children, Bristol


### A CASE OF ATAXIA IN A CHILD AGED THREE AND A HALF YEARS.

BY

EDWARD CECIL WILLIAMS, B.A., M.B. Cantab.,

*Physician to the Royal Hospital for Sick Children, Bristol.*

ATAXY may be a symptom of many conditions in children, for instance as a sequel to acute diseases—in cases of tumour of the cerebellum or pons. In the following notes I propose to describe the case of a boy aged $3\frac{1}{2}$ years, whose symptoms were observed during the first six months of his life.

H. D., aged $3\frac{1}{2}$ years, admitted into the Children's Hospital February 12th, 1911, is an illegitimate child, and his parents are first cousins. He was a full-time child, labour was normal, and he was bottle fed. During the first six months of his life it is said he was much neglected, and when adopted by his present guardian was in a very weak condition. During the first year of his life he had several attacks of bronchitis. Child was thought to be mentally deficient, did not sit up till eighteen months, and did not talk until two years of age. So far as is known has had no infectious disease. Has been under treatment in another hospital.

On admission child looked fairly healthy, there is some evidence of past rickets. He has a rather vacant expression, his head is below the average in its maximum circumference, the occipital region is rather flattened. His hair has a tendency to be coarse, and is rather dry.

Speech is indistinct, he can be taught to pronounce words properly, he does not stumble over any particular letter, he only speaks "slovenly." Palate is not high arched; teeth carious. He is not allowed to feed himself, as the movements of his arms are so unsteady, jerking his food out of the spoon, and not always succeeding in getting the spoon to his mouth. He can hyperextend his wrists to an angle of 60°. He cannot walk by himself, and throws his legs about in an ataxic manner. Knee-joints are very mobile, and can be hyperextended. He cannot stand up with his heels together with his eyes open. Knee-jerks are brisk, plantar reflex flexor. Sensation to heat and cold normal, no nystagmus, no ocular symptoms, no fundal changes. Has perfect control over his sphincters. Examination of other organs reveal no abnormality.

Since admission (two months ago) there has been a very marked improvement both in his mental and physical condition. He is much more intelligent, can learn fragments of songs, and can walk a short distance without assistance.

*Treatment.*—Small doses of thyroid extract were given, as it was thought on admission there might be some degree of thyroid insufficiency. It may be of interest here to refer to the investigation of Captain McGarrison on endemic goitre in the Chitral and Gilgit Valleys of India. During that research he came across some cases of children of goitrous parentage, whose brothers and sisters were cretins, who were the subjects of a condition called by him "a nervous form of cretinism." Those cases closely resembled cases of cerebral diplegia. They improved somewhat when treated by thyroid. Whether the improvement in this case is in any way dependent on the thyroid is a doubtful question. He is being taught to play with toys and to walk by pushing a horse on wheels around the ward.

What is the cause of the ataxy? The absence of headache, sickness and optic neuritis exclude the possibility of a cerebellar or pontine tumour. Post-diphtheritic paralysis is out of the question, there is no history of any sort of infectious disease.

Then there is Friedreich's or hereditary ataxy, in which the disease often comes on during early life, after one of the specific fevers. It is usually hereditary, and occurs in families where there is some tendency to some form of nervous disease. In Friedreich's ataxy the patient tends to get progressively worse, whereas this boy is showing signs of improvement after a comparatively short stay in hospital. I am therefore inclined to place this boy in the same category as those cases of congenital ataxia described by Batten.[1]

The morbid anatomy is at present unknown, there is much to suggest a faulty development in the cerebellum, and in some of the cases, my own being a case in point, the alteration in mental condition would suggest a probable change in the cerebrum also. It seems likely extensive histological changes will be found in the brains of these cases.

P.S.—May 15th, 1911. Boy continues to make great improvement both in intelligence and movements.

### Progress of the Medical Sciences.

[1] *Brain*, 1901, xxiv. 171; *ibid.*, 1905, xxviii. 484.

